# Seasonality, temperature and pregnancy oral glucose tolerance test results in Australia

**DOI:** 10.1186/s12884-019-2413-5

**Published:** 2019-07-24

**Authors:** Eddie X. Shen, Robert G. Moses, Jeremy J.N. Oats, Julia Lowe, H. David McIntyre

**Affiliations:** 1 Faculty of Medicine, The University of Queensland, 288 Herston Road, Brisbane, Queensland 4006 Australia; 2 Illawarra and Shoalhaven Local Health District, Wollongong Hospital, Loftus Street, Wollongong, New South Wales 2500 Australia; 3 Melbourne School of Global and Population Health, University of Melbourne, Parkville, Victoria 3010 Australia; 4 University of Toronto, 27 King’s College Circle, Toronto, Ontario M5S 1A1 Canada; 5 Mater Research, Level 3, Aubigny Place, Raymond Terrace, Brisbane, Queensland 4101 Australia

**Keywords:** Gestational diabetes mellitus, Oral glucose tolerance testing, Seasonal variability, HAPO, Fetal outcomes

## Abstract

**Background:**

The oral glucose-tolerance test (OGTT) is currently the standard method for diagnosis of gestational diabetes (GDM). We conducted a post hoc analysis using the Australian Hyperglycemia and Adverse Pregnancy Outcome (HAPO) data to determine seasonal variations in OGTT results, the consequent prevalence of GDM, and association with select perinatal parameters.

**Method:**

Women enrolled in the Australian HAPO study sites (Brisbane and Newcastle) from 2001 to 2006 were included if OGTT results between 24 to 32 weeks gestation were available (*n* = 2120). Fasting plasma glucose, 1-h plasma glucose, 2-h plasma glucose, HbA1c, HOMA-IR, and umbilical cord C-peptide and glucose values were categorized by season and correlated to monthly temperature records from the Australian Bureau of Meteorology for Brisbane and Newcastle. GDM was defined post hoc using the IADPSG/WHO criteria.

**Results:**

Small but significant (*p* <  0.01 on ANOVA) elevations in fasting glucose (+ 0.12 mM), HbA1c (+ 0.09%), and HOMA-IR (+ 0.88 units) were observed during the winter months. Conversely, higher 1-h (+ 0.19 mM) and 2-h (+ 0.33 mM) post-load glucose values (both *p* <  0.01) were observed during the summer months. The correlations between fasting glucose, 1-h glucose, 2-h glucose, and HbA1c with average monthly temperatures confirmed this trend, with positive Pearson’s correlations between 1-h and 2-h glucose with increasing average monthly temperatures, and negative correlations with fasting glucose and HbA1c. Further, umbilical cord C-peptide and glucose displayed negative Pearson’s correlation with average monthly temperature, aligned with trends seen in the fasting plasma glucose. Overall prevalence of GDM did not display significant seasonal variations due to the opposing trends seen in the fasting versus 1-h and 2-h post-load values.

**Conclusion:**

A significant winter increase was observed for fasting plasma glucose, HbA1c, and HOMA-IR, which contrasted with changes in 1-h and 2-h post-load venous plasma glucose values. Interestingly, umbilical cord C-peptide and glucose displayed similar trends to that of the fasting plasma glucose. While overall prevalence of GDM did not vary significantly by seasons, this study illustrates that seasonality is indeed an additional factor when interpreting OGTT results for the diagnosis of GDM and provides new direction for future research into the seasonal adjustment of OGTT results.

## Introduction

Gestational diabetes mellitus (GDM) is the most common medical problem encountered during pregnancy. GDM prevalence varies widely depending on the diagnostic criteria used and the risk factors present in the population being examined. Recently, several papers have reported a seasonal variation in the prevalence of GDM with higher rates in summer and lower rates in winter. These findings have been reported in both the northern and the southern hemispheres, in places that are temperate or have a wide fluctuation in ambient temperatures, and with different diagnostic criteria [1–4].

The World Health Organization (WHO) have based their current diagnostic criteria [5] for hyperglycemia in pregnancy (HIP) on the recommendations of the International Association of Diabetes in Pregnancy Study Groups (IADPSG) [6] which in turn were based largely on the findings of the Hyperglycemia and Adverse Pregnancy Outcome (HAPO) study [7]. HAPO was an international multi-center study with glucose tolerance testing data on more than 20,000 women and detailed perinatal outcome data.

While seasonal variations in GDM have been reported, the implications of this on whether GDM is over-diagnosed in the summer or under-diagnosed in the winter is undetermined. This debate may be further informed by investigating associations with neonatal parameters which adds additional perspective to this topic. Further, the unique HAPO cohort provides centralized data with a high level of quality control, which differs from existing studies largely obtained from routine laboratory data or administrative databases. The current study aimed to investigate the association of seasonality with glucose metabolism and GDM prevalence and selected neonatal outcomes by conducting a post hoc analysis of the HAPO data for the two participating Australian centers.

## Methods

### Patient selection

All women enrolled at the Australian HAPO study sites from 2001 to 2006 were included in the current analysis if results from the oral glucose tolerance test (OGTT), performed between 24- and 32-weeks’ gestation for potential participation in the HAPO study, were available (*n* = 2120). This cohort represents 100% of all women who underwent OGTT at the Brisbane or Newcastle sites at a timepoint where no results were available, and no participants had been excluded from the study. A total of 23/2120 women (1.1%) were unblinded due to OGTT results (fasting > 5.8 or 2 h > 11.1 mmol/L), with an insignificant impact on the results. 1721 results for umbilical cord C-peptide and 1694 for cord glucose were recorded. Missing umbilical cord results were due to lack of sample collection (eg. delivery of neonate outside working hours) and are not related to maternal hyper or hypoglycemia.

### Data collection

Full methodology for the HAPO study has been previously described [7]. Briefly, each woman underwent a standard 75-g oral glucose-tolerance test (OGTT) after an overnight fast, between 24- and 32-weeks’ gestation. Venous plasma glucose (VPG) levels were taken in the fasting state and at one and two hours after the glucose load. All laboratory analyses were stored on ice and processed in an air-conditioned environment at the HAPO study central laboratory in Belfast, UK as previously described. Height and weight were measured and body mass index (BMI) calculated at the time of the OGTT visit. Ethnicity was self-reported by each participant. Glycated hemoglobin A1c (HbA1c) was measured in the fasting state and serum C-peptide levels were measured fasting and one-hour post glucose load. Fetal blood was obtained from the umbilical artery immediately following delivery. All procedures were designed to ensure extensive quality control and to reduce recruitment bias. [8]. HOMA-IR values were calculated utilising fasting VPG and fasting C-peptide values [9]. Climate date and average monthly temperature was derived from the Australian Government Bureau of Meteorology. The Brisbane station is 1.5 km away from the Mater Hospital, where all Brisbane blood samples were taken. The Newcastle station is 5.3 km away from the John Hunter Hospital at which Newcastle blood samples were taken. We do not have records of the participants’ home addresses. The average temperature for each month was recorded as a monthly maximum and minimum temperature for each site for each month of each year. The mean of these two readings was used as the monthly average daily temperature, matched for HAPO site, month and year of the OGTT collection date [10].

GDM was defined post hoc using WHO diagnostic criteria. As these analyses involve data collected at the OGTT visit, women excluded for hyper/hypoglycemia under the HAPO protocol are included in the current report.

### Statistical analysis

Data distributions were examined using PP plots and approximated normality. No data transformations were undertaken. Continuous variables are reported as mean (standard deviation). Statistical analysis was undertaken using one-way ANOVA, with season (Summer, Autumn, Winter, Spring) as the independent variable and post hoc testing used independent sample t-tests with Bonferroni corrections. Significance was accepted at the 5% level on two tailed testing. Frequencies and percentage values were recorded for all categorical variables, and significance testing was done via χ^2^ test. Pearson’s correlation coefficients were used to assess the associations between the FPG, 1-h post-load glucose, 2-h post-load glucose and HbA1c and average monthly temperatures. All statistical analysis was conducted via the SPSS Statistical package version 24.

## Results

A total of 2120 Australian women with a mean age of 29.6 ± 5.4 years were included in this study, with 1448 recruited from Brisbane, Queensland and 674 recruited from Newcastle, New South Wales. 91.4% of this cohort (*n* = 1940) were of Anglo-Saxon Caucasian descent. The mean BMI at time of OGTT was 29.1 ± 5.8 kg/m^2^. The mean age of gestation at OGTT was 28 ± 1.3 weeks. No significant associations were observed between the season of the OGTT and maternal age, maternal BMI or gestational age at OGTT (Table 1). Given Australia’s seasonal differences, December, January, and February were classified as summer, March, April, May were autumn, June, July, and August were classified as winter, and September, October, and November were spring. Average monthly temperatures ranged from a high of 27.0 °C in January to a low of 13.1 °C in June. Mean average monthly temperature was 25.3 °C in the summer months, 21.0 °C in autumn, 20.6 °C in spring, and 15.4 °C in the winter months. We found mean fasting VPG to be lowest in the summer months, and highest in the winter with statistical significance (Table 2). A similar trend can be seen in the mean HbA1c and HOMA-IR values. Conversely, the mean 1 and 2-h post-OGTT values peaked in the summer months and were lowest in the winter. Further details on mean fasting, 1-h, and 2-h VPG as well as HbA1c and HOMA-IR values by seasons are presented in Table 2. Notably, we found the greatest difference in 1-h and 2-h glucose values to be between summer and winter (Table 3). Similarly, the prevalence of GDM based on fasting VPG was greatest in the winter, although not statistically significant. The opposite trend is seen on 1-h, or 2-h values. Interestingly, GDM prevalence by 2-h values were higher in the spring than the summer. No significant difference in the overall prevalence of GDM by seasons were observed. Further details on GDM prevalence based on the WHO criteria (5) between seasons can be seen in Table 4. The correlations between fasting VPG, 1-h post-OGTT VPG, 2-h post-OGTT VPG, and HbA1c between monthly average temperatures can be seen in Table 5 and follows a similar trend to that observed by seasons. Lastly, the role of temperature on neonatal glucose metabolism can be seen in Tables 6 and 7. While the seasonal difference between means did not meet significance cut off, we found the correlation between umbilical cord C-peptide and glucose with mean temperature at birth in the same direction as the fasting VPG and HbA1c with average monthly temperature at OGTT.

**Table 1:** Participant characteristics by season

	Summer (SD)	Autumn (SD)	Winter (SD)	Spring (SD)	*p* value
Mean age (years)	29.6 (5.3)	29.4 (5.3)	29.4 (5.5)	29.5 (5.4)	= 0.953
Anglo-Saxon descent	90.8%	92.1%	91.2%	91.6%	= 0.895
Mean BMI (kg/m^2^)	29.1 (6.0)	29.1 (5.7)	29.4 (5.8)	29.1 (5.8)	= 0.741
Mean gestational age (weeks)	28.2 (1.5)	28.1 (1.4)	28.2 (1.2))	28.1 (1.3)	= 0.8316

Data represented as mean (SD) or %

**Table 2:** Mean fasting, 1-h post-load and 2-h post-load VPG, HbA1c, and HOMA-IR values by season

	Summer (SD) mmol/L *n* = 476	Autumn (SD) mmol/L *n* = 530	Winter (SD) mmol/L *n* = 558	Spring (SD) mmol/L *n* = 558	*p* value
Fasting plasma glucose	4.4 (0.4)	4.5 (0.4)	4.5 (0.4)	4.5 (0.4)	< 0.001
1-h plasma glucose	7.6 (1.7)	7.4 (1.5)	7.4 (1.5)	7.4 (1.6)	= 0.002
2-h plasma glucose	6.4 (1.3)	6.3 (1.3)	6.1 (1.2)	6.3 (1.4)	< 0.001
HbA1c	4.7 (0.3)	4.8 (0.4)	4.8 (0.4)	4.7 (0.4)	< 0.001
HOMA-IR	7.5 (4.0)	8.1 (4.4)	8.4 (4.5)	8.1 (4.6)	= 0.014

Data represented as mean (SD) or %

**Table 3:** Comparison of 1-h and 2-h plasma glucose values by individual seasons

1-h plasma glucose	Autumn	Spring	= 1
		Summer	= 0.376
		Winter	= 0.308
	Spring	Autumn	= 1
		Summer	= 0.504
		Winter	= 0.196
	Summer	Autumn	= 0.376
		Spring	= 0.504
		Winter	= 0.001
	Winter	Autumn	= 0.308
		Spring	= 0.196
		Summer	= 0.001
2-h plasma glucose	Autumn	Spring	= 1
		Summer	= 0.491
		Winter	= 0.110
	Spring	Autumn	= 1
		Summer	= 1
		Winter	= 0.012
	Summer	Autumn	= 0.491
		Spring	= 1
		Winter	< 0.0001
	Winter	Autumn	= 0.110
		Spring	= 0.012
		Summer	< 0.0001

**Table 4:** Prevalence of GDM by season. Prevalence of GDM with 1-h and 2-h post-OGTT VPG values excludes those with an abnormal fasting value

	Summer (%) *n* = 476	Autumn (%) *n* = 530	Winter (%) *n* = 558	Spring (%) *n* = 558	*p* value
GDM prevalence by fasting plasma glucose ≥5.1 mmol/L *n* = 79	5.5%	6.4%	9.5%	8.4%	= 0.055
GDM prevalence by 1-h post-load glucose ≥10.0 mmol/L *n* = 31	6.5%	3.6%	2.5%	3.2%	= 0.009
GDM prevalence by 2-h post-load glucose ≥8.5 mmol/L *n* = 12	4.2%	3.2%	1.6%	4.7%	= 0.033
Overall GDM prevalence by WHO criteria *n* = 122	13.9%	12.1%	12.9%	15.1%	= 0.510

Data represented as %

**Table 5:** Correlation of average monthly temperature at time of OGTT to fasting plasma glucose, 1-h post-OGTT plasma glucose, 2-h post-OGTT plasma glucose, and HbA1c

Correlation with average monthly temperature	Pearson’s r	*p* value
Fasting plasma glucose	- 0.145 (−0.187, − 0.103)	< 0.001
1-h plasma glucose	+ 0.079 (+ 0.036, + 0.120)	< 0.001
2-h plasma glucose	+ 0.093 (+ 0.051, + 0.135)	< 0.001
HbA1c	- 0.069 (−0.113, − 0.025)	= 0.002

**Table 6:** Cord C-peptide and cord plasma glucose values by season

	Summer (SD) mmol/L *n* = 476	Autumn (SD) mmol/L *n* = 530	Winter (SD) mmol/L *n* = 558	Spring (SD) mmol/L *n* = 558	*p value*
Umbilical cord C-peptide	0.993 (0.557)	1.062 (0.661)	1.076 (0.646)	0.996 (0.538)	= 0.087
Umbilical cord glucose	4.658 (1.180)	4.754 (1.187)	4.758 (1.219)	4.578 (1.096)	= 0.066

Data represented as mean (SD)

**Table 7:** Correlation of average monthly temperature at birth to cord C-peptide and cord glucose

Correlation with average monthly temperature	Pearson’s r	*p* value
Umbilical cord C-peptide (*n = 1721*)	- 0.153 (−0.199, −0.107)	< 0.001
Umbilical cord glucose (*n = 1694*)	- 0.126 (−0.172, − 0.079)	< 0.001

## Discussion

We demonstrated a small but statistically significant seasonal variation in fasting VPG, HbA1c, and HOMA-IR (higher in winter) contrasted with 1-h and 2-h post-OGTT VPG (higher in summer). This association was confirmed by significant negative correlations between fasting VPG, HbA1c and HOMA-IR and average monthly temperature again contrasting with positive correlations between 1-h and 2-h post-OGTT VPG with average monthly temperature. The finding of higher mean 1-h and 2-h post-OGTT VPG in summer is consistent with previous studies [1–3, 11, 12], indicating a higher prevalence of GDM by those measures. However, the findings of higher fasting VPG, HbA1c and HOMA-IR in winter and with lower temperatures differ from the majority of previous reports but have been shown in some settings [13, 14].

In our cohort, due to the opposing directions of changes in the fasting versus post-OGTT VPG values, the overall prevalence of GDM based on the new WHO criteria was similar in the summer and winter months and did not significantly vary by season. In contrast, previous literature suggests a positive correlation between increasing temperatures and overall GDM prevalence [4]. While our findings differ from previous major reports, this may reflect the use of a new diagnostic criteria with lower threshold values. In example, Verburg et al., while finding overall prevalence of GDM to be greatest when tested in the summer months (correlating to an estimated date of conception in the winter), initial GDM screening was conducted via a 50 g glucose challenge test, with an abnormal threshold at > 7.8 mmol/L [4]. Further, the HAPO cohort contains centrally analysed data in a single center in Belfast, with samples stored on ice and processed in climate-controlled laboratories. This contrasts to previous Australian studies which found a variation in GDM prevalence with season, however largely measured ambient temperature or were not climate controlled [1, 15]. Further supporting this point, Moses et al. (1995) found that, under an air conditioned environment, GDM prevalence did not significantly vary by season [11]. Lastly, we noted a negative Pearson’s correlation between umbilical cord C-peptide and cord glucose with temperature at birth, consistent with the correlations at OGTT with fasting VPG and HbA1c. While this data does not prove causality regarding potential effects on fetal outcomes, it does provide a novel perspective to the existing literature, as so far, no study has examined neonatal parameters in this context.

### Seasonal variations in fasting versus postprandial glucose values

The opposing trends seen between fasting VPG, HbA1c, HOMA-IR, and post-OGTT VPG may reflect differing underlying (patho) physiology. Elevated post-OGTT VPG may be attributed to a physiological adaptation to temperature changes. Previous studies have suggested that a fluctuation of apparent glucose tolerance (as measured by post-OGTT values) with ambient temperature could be due to a peripheral redistribution of blood flow between cutaneous and visceral vascular beds driven by a rise in core body temperature, resulting in less peripheral glucose extraction and higher VPG value [15]. Alternatively, increased food intake may accompany Christmas vacation during the Australian summer months [16], though maternal weight and BMI did not differ between seasons. On the contrary, elevations in fasting VPG have been correlated to low serum vitamin D levels (as is common in colder climates), suggesting that an early gestation vitamin D deficiency alters glucose metabolism in later gestation [13]. A high fasting VPG may also relate to circannual rhythms of melatonin, which exhibits direct inhibitory effects on insulin secretion [13]. This is consistent with impaired fasting VPG reflecting a picture of declining β-cell function, as an inadequate insulin response is mounted to counteract gluconeogenic activity of the liver [17]. Conversely, post-OGTT hyperglycemia may better reflect early impaired glucose tolerance, while maintaining plasma glucose levels in the fasting state [17, 18].

### Role of seasonality on adverse neonatal outcomes

While previous studies have suggested an association between GDM prevalence and seasonality, neonatal glucose metabolism has never been examined in this context. The observed trend towards elevated cord C-peptide and glucose relating to the OGTT temperature and potentially to consequent higher glucose exposure (fasting glucose and HbA1c) is a novel finding with potential utility in GDM diagnosis. Both neonatal hyperglycemia and elevated C-peptide have been associated with increased fat mass, excessive neonatal growth, and increased skin fold measurements [19, 20]. While we are unable to demonstrate a direct correlation, we note these parameters which suggest an impaired neonatal glucose metabolism correlated with temperature in the same directional trend as an impaired fasting VPG did with temperature. Existing literature suggests a stronger role of postprandial glucose on neonatal outcomes, however these included predominantly women with pre-existing diabetes. Two previous studies highlighted closer correlation of adverse outcomes with fasting glucose in women without pre-existing diabetes, concordant with our data [20, 21]. This suggest that postprandial glucose levels may be more associated with adverse neonatal outcomes in those with overt or insulin dependent diabetes, due to an increased glucose excursion after meals, while fasting values may be more predictive of adverse neonatal outcomes in the GDM range of hyperglycemia, as a function of a defect in basal blood glucose homeostasis [20]. Overall, despite opposing trends in the fasting and post-OGTT VPG values, an elevated fasting VPG may play a more direct role on neonatal hyperinsulinemia and glucose intolerance, and potentially holds clinical significance in the diagnosis of GDM using the IADPSG/WHO diagnostic criteria, reflecting the lowered diagnostic cut-off.

### Limitations of study

The post- hoc nature of this study confers several limitations to the study results. Firstly, average monthly temperature was used, and we were unable to obtain data on the outdoor temperature on the day of each OGTT. While it is unclear precisely which temperature measurement is most relevant, it is unlikely to be the temperature on the day of the OGTT, as the OGTT was conducted indoors in a centralized climate-controlled environment. Secondly, the usage of the specific HAPO population may have introduced selection bias into the study outcomes, due to the application of inclusion and exclusion criteria, and may not represented an unbiased sample of all parous women within each surrounding area. Lastly, we did not adjust for confounding factors such as age, BMI, or parity in our analysis, and future prospective studies are needed to better clarify the associations made in this paper.

## Conclusion

Despite recent changes to the diagnostic criteria of GDM with the widespread adoption of the IADPSG/WHO diagnostic criteria, debate continues regarding optimal OGTT parameters in diagnosing GDM. The present data confirms some of the trends established by previous groups regarding the role of seasonality on blood glucose. Seasonality is indeed an additional factor in interpreting OGTT results, although the effect size is small. While it is difficult to conclude as to whether these trends indicate an over-diagnosis of GDM in the summer, or an under-diagnosis in the winter, the relationship between seasonal OGTT temperature and neonatal glucose parameters suggests a functional connection. Overall, the current data raise further questions as to our understanding of the (patho) physiology of glucose metabolism in pregnancy and consequent diagnosis of GDM and provides some new directions for future research. The results of this and previous studies may imply the need for consideration of seasonal adjustments to diagnostic glucose levels.

## Data Availability

The dataset analysed within this current study are available from the corresponding author upon reasonable request. This dataset is the property of the corresponding author, as a part of the original HAPO study. Meteorological data used in analysis during this current study are publicly available in the Australian Bureau of Meteorology repository, www.bom.gov.au.

## Abbreviations

BMI: Body Mass Index
GDM: gestational diabetes mellitus
HAPO: Hyperglycemia and Adverse Pregnancy Outcomes
HbA1c: glycated haemoglobin A1c
HIP: Hyperglycemia in pregnancy
IADPSG: International Association of Diabetes and Pregnancy Study Groups
OGTT: Oral glucose tolerance test
VPG: Venous plasma glucose
WHO: World Health Organisation
